# Genetic and environmental aetiologies of associations between dispositional mindfulness and ADHD traits: a population-based twin study

**DOI:** 10.1007/s00787-019-01279-8

**Published:** 2019-02-13

**Authors:** Nienke M. Siebelink, Philip Asherson, Elena Antonova, Susan M. Bögels, Anne E. Speckens, Jan K. Buitelaar, Corina U. Greven

**Affiliations:** 1 Department of Cognitive Neuroscience, Donders Institute for Brain, Cognition and Behaviour, Radboud University Medical Centre, Nijmegen, The Netherlands; 2 Karakter, Child and Adolescent Psychiatry, University Center, Reinier Postlaan 12, 6525 GC Nijmegen, The Netherlands; 3 Social, Genetic and Developmental Psychiatry, King’s College London, Institute of Psychiatry, Psychology and Neuroscience, London, UK; 4 Department of Psychology, King’s College London, Institute of Psychiatry, Psychology and Neuroscience, London, UK; 5 Department of Developmental Psychology, University of Amsterdam, Amsterdam, The Netherlands; 6 Research Institute of Child Development and Education, University of Amsterdam, Amsterdam, The Netherlands; 7 Department of Psychiatry, Radboudumc Centre for Mindfulness, Radboud University Medical Centre, Nijmegen, The Netherlands

**Keywords:** Attention-deficit disorder with hyperactivity, Mindfulness, Attention, Genetics, Twin study

## Abstract

To get additional insight into the phenotype of attentional problems, we examined to what extent genetic and environmental factors explain covariation between lack of dispositional mindfulness and attention-deficit/hyperactivity disorder (ADHD) traits in youth, and explored the incremental validity of these constructs in predicting life satisfaction. We used data from a UK population-representative sample of adolescent twins (*N* = 1092 pairs) on lack of dispositional mindfulness [Mindful Attention Awareness Scale (MAAS)], ADHD traits [Conners’ Parent Rating Scale-Revised (CPRS-R): inattentive (INATT) and hyperactivity/impulsivity (HYP/IMP) symptom dimensions] and life satisfaction (Students’ Life Satisfaction Scale). Twin model fitting analyses were conducted. Phenotypic correlations (*r*_p_) between MAAS and CPRS-R (INATT: *r*_p_ = 0.18, HYP/IMP: *r*_p_ = 0.13) were small, but significant and largely explained by shared genes for INATT (% *r*_p_ INATT–MAAS due to genes: 93%, genetic correlation *r*_*A*_ = 0.37) and HYP/IMP (% *r*_p_ HYP/IMP–MAAS due to genes: 81%; genetic correlation *r*_*A*_ = 0.21) with no significant contribution of environmental factors. MAAS, INATT and HYP/IMP significantly and independently predicted life satisfaction. Lack of dispositional mindfulness, assessed as self-reported perceived lapses of attention (MAAS), taps into an aspect of attentional functioning that is phenotypically and genetically distinct from parent-rated ADHD traits. The clinically relevant incremental validity of both scales implicates that MAAS could be used to explore the underlying mechanisms of an aspect of attentional functioning that uniquely affects life satisfaction and is not captured by DSM-based ADHD scales. Further future research could identify if lack of dispositional mindfulness and high ADHD traits can be targeted by different therapeutic approaches resulting in different effects on life satisfaction.

**Electronic supplementary material:**

The online version of this article (10.1007/s00787-019-01279-8) contains supplementary material, which is available to authorized users.

## Introduction

Mindfulness has been defined as the self-regulation of attention towards the present moment and the orientation to one’s experience with an attitude of curiosity, openness and acceptance [1]. Self-regulation of attention might be particularly difficult for people with attention-deficit/hyperactivity disorder (ADHD). ADHD is a heritable neurodevelopmental disorder characterised by impairing symptoms of inattention (INATT) and hyperactivity–impulsivity (HYP/IMP), commonly diagnosed according to criteria described in the Diagnostic and Statistical Manual of Mental disorders (DSM) [2]. ADHD has a prevalence of about 5% in children and adolescents and 2.5% in adults [3]. Especially for adolescents with ADHD, it is important to develop self-regulation skills and awareness of their own functioning in the transition to adulthood [4]. Mindfulness-based interventions (MBIs) [5] might target these needs and are increasingly gaining ground as an intervention for ADHD [6, 7]. Studying aspects of self-regulation captured by the concept of dispositional mindfulness in relation to ADHD traits could give additional insight into the phenotype of attentional problems and new approaches for interventions like MBIs.

Dispositional mindfulness refers to mindfulness as a psychological trait independent of mindfulness acquired as a skill through training and practice, such as meditation. This means that dispositional mindfulness can be assessed in meditation-naïve individuals, but dispositional mindfulness can also increase following mindfulness training and practice [8, 9]. The Mindfulness Attention Awareness Scale (MAAS, [10]) is a commonly used measure assessing a lack of dispositional mindfulness by experienced attention lapses in daily life and the tendency to run on “automatic pilot”. MAAS exhibits adequate psychometric properties and theoretically consistent relationships to brain activity, MBI outcomes, and mediation of MBI effects [8, 10–14]. The instrument taps into one aspect of dispositional mindfulness: (lack of) ‘attention towards the present moment’. The ‘orientation to one’s experience with an attitude of curiosity, openness and acceptance’ is not captured by MAAS. Therefore, MAAS seems closely related to constructs like inattention and inattentiveness. However, only few studies have examined relations between the lack of dispositional mindfulness and ADHD diagnosis or traits.

There is increasing evidence that the lack of dispositional mindfulness is associated with ADHD. Higher scores on dimensional assessments of ADHD traits have been associated with the lack of dispositional mindfulness (MAAS) both in university students with and without ADHD [15], as well as in high school attendees [16]. In addition, ADHD diagnosis had a strong negative association with dispositional mindfulness as assessed with the Kentucky Inventory of Mindfulness Scale (KIMS) in an adult sample of parents of children with ADHD, half of whom had a lifetime diagnosis of ADHD themselves [17]. This association between ADHD and mindfulness was largely ascribed to the KIMS subscale *acting*-*with*-*awareness,* which is closely related to MAAS [18]. Similarly, the *acting*-*with*-*awareness* subscale of the five facet mindfulness questionnaire (FFMQ) [19], comprised of MAAS and KIMS items, showed the strongest association, while the other four FFMQ subscales (*observing*, *describing*, *non*-*judging* and *non*-*reacting*) showed small or non-significant associations with ADHD outcomes in college students, of whom half had ADHD [20] and in adults with ADHD [21]. Thus, previous research suggests a negative association between ADHD symptomatology and specifically the attentional aspect of dispositional mindfulness. Therefore, it is interesting to further explore the relation between ADHD traits and experienced lapses of attention as a measure of a lack of dispositional mindfulness.

The negative association between ADHD and the attentional aspect of dispositional mindfulness may arise from aetiological overlap in the constructs to assess ADHD symptoms/traits and (a lack of) dispositional mindfulness. Both traits are heritable: a lack of dispositional mindfulness (MAAS) around 30% [22] and ADHD around 60–80% [3, 23, 24]. Further, both traits show little or no evidence for the influence of environmental risks factors shared by siblings [22, 25]. However, the genetic and environmental contributions to the association between a lack of dispositional mindfulness and ADHD have not previously been studied.

A way to explore the relevance of studying similarities and differences between these two associated concepts is to look at their incremental validity in predicting a clinically relevant outcome. ADHD traits are a description of observed behavioural inattention, hyperactivity and impulsivity, which is conceptually different from MAAS that captures perceived lapses of attention. Both ADHD traits and MAAS can predict aspects of well-being [10, 26]. In a Chinese high school population, correlations between MAAS and well-being variables remained significant when controlling for ADHD traits [16]. This suggests incremental validity of MAAS in predicting health outcomes beyond ADHD traits, showing that the scales have a complementary value, despite phenotypic overlap. However, Black, et al. [16] did not examine INATT and HYP/IMP separately.

The purpose of the present study is to examine associations between two possibly complementary attentional constructs: a lack of dispositional mindfulness and ADHD traits, in a UK population-representative sample of adolescent twins (*N* = 1092). We expect a greater shared genetic rather than environmental influence in explaining the association between the constructs. We assess the lack of dispositional mindfulness by an abbreviated 5-item version of MAAS [12], which was shown to be well-understood by adolescents in previous research and yielded meaningful associations with clinically relevant measures in relation to shared genes/environments (e.g., [22]). ADHD traits were assessed by parent report which is considered more valid as a measure of observed behaviours than self-report in youth [27, 28]. We focus on ADHD as a continuous trait, given considerable evidence that the disorder reflects the extreme of continuous traits of inattentiveness and hyperactivity/impulsivity in the general population [29, 30]. The two dimensions of ADHD are studied separately, since they show significant unique, as well as shared, genetic effects [31].

### Aims of the study

The aims of the study are, first, to examine phenotypic associations between a lack of dispositional mindfulness and ADHD traits, separately for inattentiveness and hyperactivity/impulsivity. Second, we aim to investigate the extent to which shared genetic and environmental factors explain the associations between these traits. Third, we intend to explore the incremental validity of the lack of dispositional mindfulness and ADHD traits, through studying their unique contributions to predict life satisfaction as a clinically relevant outcome.

## Method

### Sample and procedure

Data came from the 16-year assessment wave of the UK population-representative Twins Early Development Study (TEDS) [32], which consists of twins born in England and Wales between 1994 and 1996 identified through birth records (see Online Resource 1 for representativeness). In the first cohort of this wave, a scale to assess the lack of dispositional mindfulness (MAAS, 5-item version) was included in the test battery. Data for the current study were collected in spring 2011. Informed consent from parents and twins and ethical approval were obtained (PNM/09/10-104 approved by the KCL Research Ethics Committee). Families were excluded following severe pre- or perinatal complications, a severe medical condition (e.g., a chromosomal disorder, brain damage, global developmental delay, autism, blindness) or if sex or zygosity were uncertain. The final sample consisted of *N* = 1092 monozygotic (MZ) and dizygotic (DZ) twin pairs (mean age = 16.89 years, SD = 0.23, range 16.49–18.76): 418 MZ (139 males, 279 females) and 674 DZ (134 males, 217 females, 323 opposite-sex pairs).

### Measures

In the current study, Cronbach’s alpha internal consistency was acceptable (*α* = 0.76) to excellent (*α* = 0.91) for all scales (Online Resource 2).

#### The lack of dispositional mindfulness

The lack of dispositional mindfulness was assessed using an abbreviated version of MAAS, comprised of five items shown to have the highest differential item functioning [12, 33]. The 5-item version has a strong positive correlation of *r* = 0.93, *p* < 0.001, 95% CI (0.92, 0.94) [12] with the original 15-item MAAS [10]. The adolescents rated themselves on a 6-point Likert scale from 0 ‘almost never’ to 5 ‘almost always’. Higher scores reflect more experienced lapses of attention.

#### ADHD traits

ADHD traits were assessed using parent ratings on the DSM-IV-based ADHD subscales of the Revised Conners’ Parent Rating Scale (CPRS-R) [34], which consist of a 9-item INATT subscale and a 9-item HYP/IMP subscale. Parents rated the behaviour of their children on a 4-point Likert scale from 0 ‘not true at all’ to 3 ‘definitely true’. Higher scores reflect a higher level of ADHD traits.

#### Life satisfaction

Life satisfaction, as a primary component of subjective well-being [35], was assessed using self-rating on the shortened 21-item Students’ Life Satisfaction Scale [36]. Adolescents rated themselves on a 6-point Likert scale from 1 ‘strongly agree’ to 6 ‘strongly disagree’.

### Statistical analyses

Analyses were based on the Twin Method [37], which allows estimating the relative contributions of additive genetic (heritability, *h*^2^ or *A*), shared environmental (*C*), and non-shared environmental (*E*) influences on a trait or the covariation between traits. *E* includes measurement error.

To study phenotypic (observed) associations (*r*_p_) between MAAS and ADHD traits (INATT and HYP/IMP separately) (aim 1) and to study genetic and environmental aetiologies of these associations (aim 2), structural equation twin model fitting was conducted in M× [38]. First, a constrained saturated model was used to derive twin and cross-twin cross-trait (CTCT) correlations. Twin correlations are within-pair within-trait correlations, that is, *r*_**MZ**_ and *r*_**DZ**_ are the correlations within, respectively, MZ and DZ pairs for one trait (MAAS, INATT or HYP/IMP). To obtain CTCT correlations, one trait (e.g., MAAS) in twin 1 is correlated with another trait (e.g., INATT) in the co-twin. Twin and CTCT correlations allow a first impression of the extent to which individual differences (variance) in variables, and their associations (covariation), are attributable to genetic (*A*) and environmental (*C* and *E*) factors.

**Fig. 1:**
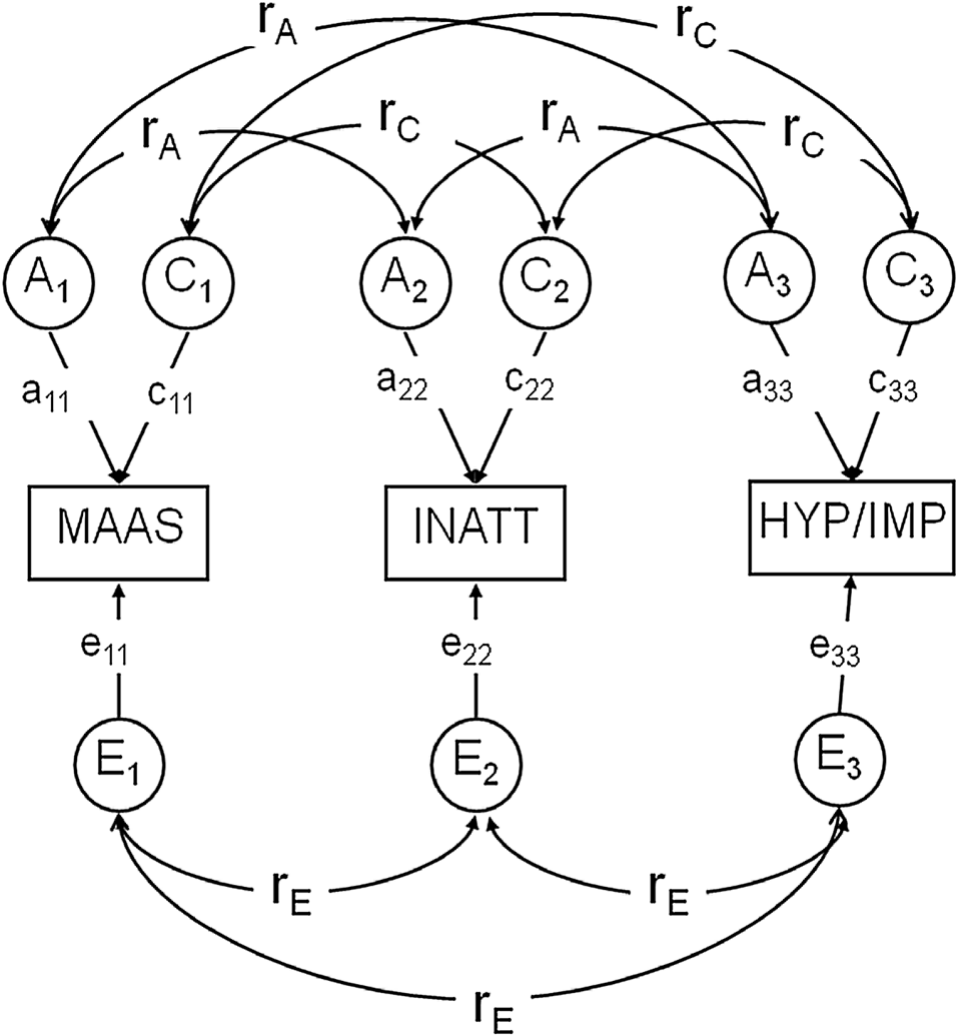
Trivariate ACE model. Rectangles refer to the variance of observed variables. *HYP/IMP* hyperactivity–impulsivity, *INATT* inattentiveness, *MAAS* Mindful Attention Awareness Scale. Circles refer to latent genetic (*A*), shared environmental (*C*) and non-shared environmental (*E*) factors. Each latent variable has a variance of 1. The curved double-headed arrows refer to genetic and environmental correlations (*r*_*A*_, *r*_*C*_, *r*_*E*_)

Next, these impressions were confirmed by fitting a Cholesky decomposition, represented as a correlated factors solution (Fig. 1), which facilitates the estimation of *r*_p_, *A*, *C* and *E* influences, and genetic and environmental correlations (*r*_*A*_, *r*_*C*_, *r*_*E*_) between MAAS and CPRS-R dimensions. These correlations can range from − 1 to 1, and indicate the extent of genetic and environmental sharing between two traits.

We also estimated the proportion of the phenotypic correlations attributable to genes or environments. For example, from Fig. 1, the proportion of *r*_p_ between MAAS and INATT due to *A*, can be estimated as *r*_*A*_ × √*a*_11_ × √*a*_22_ divided by *r*_p_. Previous studies using the current sample [22, 39] revealed no aetiological sex differences for MAAS and CPRS-R, and hence these are not modelled in the present study.

The Conners’ scales were positively skewed and transformed using a Van der Waerden transformation [40]. Following standard procedures, measures were regressed for sex and age [41], and residual scores were included in the analysis. Full information maximum likelihood estimation was used to handle missing data. Likelihood-based 95% confidence intervals (CIs) were obtained to inform the precision of parameter estimates, which presents an advantage over using standard errors in structural equation twin models [42]. CIs crossing zero indicate non-significance of an estimate. CIs that do not overlap indicate two estimates differ significantly. Akaike’s information criterion (AIC, [43]) and Bayesian information criterion (BIC, [44]) were used to compare the fit of the ACE model to a fully unconstrained saturated model. The best combination of goodness-of-fit and parsimony is achieved by the model with the lowest AIC and BIC values.

To address the incremental validity of MAAS and ADHD traits (aim 3), regression analyses were conducted using Stata [45]. The ‘cluster’ command was used which takes into account the non-independence of twin data by calculating robust standard errors [45]. The predictor (either MAAS, INATT or HYP/IMP, regressed for age and sex) was entered in the first step and the other predictor (INATT, HYP/IMP or MAAS) in the second step of the regression model. This allows examining if MAAS explains additional variance (*R*^2^) from step 1 to step 2 in the dependent variable (life satisfaction) not accounted for by ADHD traits and vice versa.

## Results

The descriptive statistics for all measures are presented in the Online Resource 2.

### Phenotypic, twin and cross-twin cross-trait correlations

The MAAS showed small but significant phenotypic correlations with INATT (*r*_p_= 0.18, 95% CI 0.13–0.22) and HYP/IMP (*r*_p_ = 0.13, 95% CI 0.08–0.17), with the lack of dispositional mindfulness reflecting higher ADHD traits (Table 1). The phenotypic correlation between MAAS and INATT was not significantly larger than between MAAS and HYP/IMP.

**Table 1:** Phenotypic and cross-twin cross-trait (CTCT) correlations between self-rated MAAS and parent-rated ADHD traits

	MAAS	INATT	HYP/IMP
Phenotypic correlations			
MAAS	–		
INATT	0.18 (0.13–0.22)	–	
HYP/IMP	0.13 (0.08–0.17)	0.49 (0.45–0.52)	–
MZ and DZ twin correlations			
MZ	0.37 (0.28–0.45)	0.78 (0.74–0.81)	0.86 (0.83–0.88)
DZ	0.15 (0.08–0.23)	0.45 (0.39–0.51)	0.53 (0.48–0.59)
MZ (below diagonal) and DZ (above diagonal) CTCT correlations			
MAAS	–	0.07 (0.02–0.12)	0.06 (0.01–0.11)
INATT	0.16 (0.11–0.22)	–	0.38 (0.33–0.43)
HYP/IMP	0.10 (0.05–0.15)	0.46 (0.42–0.50)	–

95% confidence intervals in parentheses

*ADHD* attention-deficit/hyperactivity disorder, *HYP/IMP* hyperactivity–impulsivity, *INATT* inattentiveness, *MAAS* Mindful Attention Awareness Scale, *CTCT* cross-twin cross-trait, *MZ* monozygotic, *DZ* dizygotic

The MZ twin correlations were larger than the DZ correlations, but less than one (Table 1), indicating the presence of additive genetic (*A*) and non-shared environmental (*E*) influences on MAAS, INATT and HYP/IMP. For MAAS, the DZ correlation was slightly less than half the MZ correlation, suggesting the absence of shared environmental effects (*A*, *E* rather than *A*, *C*, *E*), and that heritability should be interpreted as both additive (sum of the effects of the individual alleles at all loci that influence the trait) and dominant (interactions between alleles at the same locus) genetic effects. For ADHD dimensions, DZ twin correlations were greater than half the MZ correlations (Table 1), indicating some influence of shared environment (*C*).

The MZ CTCT correlations were larger than the DZ CTCT correlations (Table 1), suggesting a role for additive genetic factors (*A*) in association between MAAS and ADHD dimensions. The MZ CTCT correlations (MAAS–INATT = 0.16; MAAS–HYP/IMP = 0.10) were similar in magnitude to the phenotypic correlations (MAAS–INATT = 0.18; MAAS–HYP/IMP = 0.13) (Table 1), suggesting non-shared environments (*E*) played only a small role in explaining association between MAAS and ADHD traits. DZ CTCT correlations (MAAS–INATT = 0.07; MAAS–HYP/IMP = 0.06) were roughly half the respective MZ CTCT correlations, suggesting shared environmental (*C*) influences play no or little role in association between MAAS and ADHD traits.

### ACE model results

The ACE model was a good fit to the data as indicated by the negative AIC and BIC values: *χ*^2^ (*df* = 111) = 189.78, *p* < 0.001, AIC = − 32.22, BIC = − 293.37. As dropping parameters can artificially inflate non-significant estimates, results from the full ACE model are presented. Significant heritability was found for MAAS (*A*= 35%), INATT (*A*= 61%) and HYP/IMP (*A*= 65%) (Table 2). The remainder of variance was completely due to non-shared environments for MAAS (*C*= 0%, *E*= 65%) and due to both shared and non-shared environmental influences for INATT (*C*= 18%, *E*= 21%) and HYP/IMP (*C*= 22%, *E*= 13%) (Table 2). From the aetiological correlations between MAAS and ADHD traits, only the genetic correlations were significant, with a modest overlap (*r*_*A*_= 0.37 for INATT and *r*_*A*_= 0.21 for HYP/IMP) (Table 2). The estimations of the proportion of *r*_p_ due to *A*, *C* and *E* showed that shared genetic influences largely explained the phenotypic correlations of MAAS with INATT (% *r*_p_ due to *A*= 93%, *C*= 0%, *E*= 6%) and HYP/IMP (% *r*_p_ due to *A*= 81%, *C*= 0%, *E*= 19%) (Table 2). The C path on MAAS and those connecting MAAS and ADHD dimensions were non-significant and could be dropped without a significant decrease in fit, *χ*^2^ (*df *= 3) = 0.00, *p* = 1.00.

**Table 2:** Genetic and environmental parameter estimates (on diagonals), genetic and environmental correlations (below diagonals) and proportions of phenotypic correlations due to genetic and environmental factors (above diagonals)

	MAAS	INATT	HYP/IMP
A estimates			
MAAS	**0.35 (0.17–0.42)**	93%	81%
INATT	0.37 (0.18–0.61)	**0.61 (0.51–0.70)**	52%
HYP/IMP	0.21 (0.02–0.42)	0.40 (0.31–0.48)	**0.65 (0.56–0.75)**
C estimates			
MAAS	**0.00 (0.00–0.13)**	0%	0%
INATT	0.50 (− 1.00–1.00)	**0.18 (0.10–0.27)**	41%
HYP/IMP	0.50 (− 1.00–1.00)	1.00 (0.97–1.00)	**0.22 (0.13–0.30)**
E estimates			
MAAS	**0.65 (0.58–0.73)**	6%	19%
INATT	0.03 (− 0.06–0.12)	**0.21 (0.18–0.25)**	7%
HYP/IMP	0.08 (− 0.01–0.17)	0.21 (0.11–0.30)	**0.13 (0.11–0.15)**

Results from a trivariate ACE model. Genetic (heritability A), shared environmental (C) and non-shared environmental (E) parameter estimates are presented in bold on the diagonals. Genetic and environmental correlations are given below the diagonals. Proportion of phenotypic correlations due to ACE is presented above the diagonals. 95% confidence intervals (CIs) in parentheses. CIs that cross zero indicate that the estimate is non-significant. Proportions of phenotypic correlations due to ACE are calculated as the product of the square roots of the A, C, E parameter estimates multiplied by genetic and environmental correlations, are presented above the diagonals. For example, the proportion of the phenotypic correlation (*r*_p_ = 0.18, see Table 1) between MAAS and INATT due to A was: (√0.35*√0.61*0.37)/0.18 = 93% (deviations due to rounding error). The wide CIs around shared environmental correlations with respect to MAAS suggest they cannot reliably be estimated. This is explained by the non-significant shared environmental influences on MAAS, estimated at zero. As a result, dropping all C paths for MAAS resulted in an almost identical table as the one shown here for the full model

*HYP/IMP* hyperactivity–impulsivity, *INATT* inattentiveness, *MAAS* Mindful Attention Awareness Scale

### Incremental validity

MAAS was a significant and negative predictor of life satisfaction beyond INATT and HYP/IMP and vice versa (Table 3), providing evidence for incremental validity and partly independent contributions to predicting life satisfaction. MAAS explained 3.7% of variance in life satisfaction (beyond age and sex), INATT and HYP/IMP another 3.5% and 0.2%. Likewise, INATT and HYP/IMP explained, respectively, 4.7% and 0.7% of variance in life satisfaction (beyond age and sex), MAAS another 2.5% and 3.2%.

**Table 3:** Regression analyses of MAAS and ADHD traits on life satisfaction

	*B*	*R*^2^/∆*R*^2^		*B*	*R*^2^/∆*R*^2^		*B*	*R*^2^/∆*R*^2^		*B*	*R*^2^/∆*R*^2^
Step 1:		*R*^2^ = 0.037			*R*^2^ = 0.047			*R*^2^ = 0.037			*R*^2^ = 0.007
MAAS	− 0.13***		INATT	− 0.14***		MAAS	− 0.13***		HYP/IMP	− 0.06**	
Step 2:		∆*R*^2^ = 0.035			∆*R*^2^ = 0.025			∆*R*^2^ = 0.002			∆*R*^2^ = 0.032
MAAS	− 0.11***		INATT	− 0.12***		MAAS	− 0.12***		HYP/IMP	− 0.04*	
INATT	− 0.12***		MAAS	− 0.11***		HYP/IMP	− 0.04*		MAAS	− 0.12**	

*HYP/IMP* hyperactivity–impulsivity, *INATT* inattentiveness, *MAAS* Mindful Attention Awareness Scale

*R*^2^ % of variance explained in step 1. ∆*R*^2^ incremental % of variance explained in step 2. *B* unstandardised regression coefficient. Corrections were applied for: (1) non-independence of data (‘cluster’ command in STATA); (2) multiple testing (false discovery rate, *α* at 0.05); (3) age and sex. ***p < 0.0001, **p < 0.01, *p < 0.05

## Discussion

This is the first report on the genetic and environmental aetiologies of phenotypic associations between a lack of dispositional mindfulness and the INATT and HYP/IMP dimensions of ADHD traits, allowing to explore the complementary value of these two attentional constructs in an adolescent population. We also explored if the lack of dispositional mindfulness and ADHD traits independently contribute to predicting life satisfaction. MAAS and ADHD trait measures showed small significant correlations, which were largely explained by shared genetic influences. However, genetic correlations between the lack of dispositional mindfulness and ADHD trait measures were modest and environmental correlations non-significant. In addition, the two attentional constructs, MAAS and CPRS-R, were unique negative predictors of life satisfaction, an important aspect of mental well-being, supporting the respective incremental validity of these questionnaires as clinically relevant outcomes.

The phenotypic correlations between the lack of dispositional mindfulness and ADHD traits in our study were lower than found in the previous studies. Earlier results showed larger correlations between dispositional mindfulness as assessed by MAAS and ADHD traits in high school attendees aged 14–20 years (*r* = − 0.65, *p* < 0.01) [16] and in university students aged 18–37 years with ADHD (*r* = − 0.74, *p* < 0.0001) and without ADHD (*r* = − 0.65, *p* < 0.0001) [15]. However, Black et al. [16] assessed ADHD traits with a self-report questionnaire consisting of six items of the Diagnostic Interview Schedule for Children (DIS-C) and dispositional mindfulness with a 6-item version of MAAS (same five self-report items used in present study plus one). Keith et al. [15] also used self-report to assess ADHD traits (the DSM-IV-based Adult Self-Report Scale) as well as the full (15-item) MAAS version. The use of self-assessment measures for both dispositional mindfulness and ADHD traits could therefore have contributed to the previously found higher correlations. In the current study, correlations were not confounded by shared measurement error, as ADHD traits were assessed using parent report in addition to the MAAS self-report. Dispositional mindfulness, as conceptualized by MAAS, is typically assessed using self-report, as it aims to capture awareness and perceived experiences. In contrast, parent report is considered more valid to assess ADHD traits than self-report in youth [27, 28]. The current study used a valid and reliable parent report to assess ADHD traits and it can be argued that the results give a more accurate estimation of the phenotypic association between MAAS and ADHD traits than previous studies. The overlap between the lack of dispositional mindfulness and ADHD traits might hence be smaller than previously thought.

Another finding worth noting is the absence of a significant difference between INATT and HYP/IMP in their phenotypic correlation with MAAS. Edel et al. [46] found MAAS to be correlated with only the INATT dimension (small-to-moderate, exact correlations not reported) in adults with ADHD. This could possibly be explained by the older age of their sample, because HYP/IMP tends to decline with age, whilst INATT shows a more stable trajectory [47, 48].

The results from the twin models highlight modest overlap of genetic, and no significant overlap of environmental influences. Genetic factors largely explained the phenotypic covariation between the lack of dispositional mindfulness and ADHD traits. This is in line with the “generalist genes hypothesis” [49, 50], stating that genes act in a way that they influence more than one trait, thereby accounting for associations between traits. Such genetic overlap suggests that some of the genes associated with ADHD traits are expected to play a role in the lack of dispositional mindfulness. Given the large research effort on genetic influences on ADHD [3, 51, 52], this may add to understanding the genetic aspects of dispositional mindfulness [17]. Alternative models explaining the genetic associations also need to be evaluated, such as causal or reciprocal relationship between the traits [53].

Trait-specific environmental effects indicate that the environment contributes more to differentiation among the traits rather than overlap between them. It is unknown what these influences are, but could involve differential effects of parenting, life events, divergent cultural exposure, in addition to measurement error. Reductions in ADHD traits have been found following MBIs in normally developing [54, 55] and ADHD child, adolescent and adult populations [6], partially mediated by an increase in the *acting*-*with*-*awareness* facet of KIMS [56]. Therefore, future research is needed to explore whether the role of genetic and environmental factors on the lack of dispositional mindfulness and its association with ADHD traits may change following MBI.

One proposed mechanism linking the lack of dispositional mindfulness and ADHD traits involves regulation of mind wandering which is highly correlated with MAAS [57] and with ADHD traits [58]. MBI might improve control of mind wandering by enhancing regulation of DMN deactivation (e.g., [59, 60]) and altering DMN connectivity with task positive regions, which is implicated in ADHD and associated with poorer attentional regulation [61–65]. This is supported by research showing that MBI improves meta-awareness [66] and increased meta-awareness (awareness that your mind has wandered) has been found to mediate the association of ADHD with the detrimental effects of mind wandering [67]. In addition, an RCT looking at the neurophysiological correlates of performance monitoring in ADHD also showed an increase of meta-awareness (of errors) following MBI, which was correlated with increased *acting*-*with*-*awareness* facet of FFMQ and decreased HYP/IMP symptoms [68]. The shared genetic aetiology could reflect an underlying mechanism influencing both the lack of dispositional mindfulness and ADHD traits, such as regulation of mind wandering.

However, although both adolescents with a lack of dispositional mindfulness and adolescents with high levels of ADHD traits likely experience lapses of attention, the underlying aetiologies were largely independent, suggesting different mechanisms that could underlie the expression of traits captured by MAAS and measures of ADHD traits. Furthermore, MAAS and ADHD traits were unique negative predictors of life satisfaction, replicating the findings of Black et al. [16], and extending it by showing that both INATT and HYP/IMP separately contribute to the prediction of life satisfaction. Measures based on the DSM criteria for ADHD (like CPRS-R) tap into the behavioural consequences of the attentional lapses (such as ‘often loses things’, ‘does not seem to listen’), whereas MAAS taps into potential underlying experience, i.e., lapse of attention or ‘automatic pilot’. These different aspects of attentional problems, with different impacts on life satisfaction, might benefit from different therapeutic approaches. For example, cognitive behavioural therapy for adolescents with ADHD focuses on reducing ADHD symptoms (the behavioural consequences) [69], while MBIs might target mindfulness skills (the underlying experience) [56]. Using a multidimensional approach of attention gives additional insight into effects and working mechanisms of different therapeutic interventions that can complement each other or can give direction to personalised treatment of attentional problems.

### Strengths and limitations

Strengths of the current study are the use of a large population-representative sample of twins allowing genetically informative analyses, and the use of a parent- rather than self-report measure of ADHD traits. In addition, because MAAS is self-report and ADHD traits were assessed with parent report, the current phenotypic correlations are not simply a result of shared rater variance. However, the use of different informants comes with limitations as well. The low correlations and incremental validity might be a result of informant discrepancies rather than an actual difference between the constructs. Nevertheless, in previous studies, substantially higher correlations were found between ADHD ratings by different informants compared to the phenotypic correlations found in the current study (INATT: *r*_p_ = 0.18, HYP/IMP: *r*_p_ = 0.13). In a comparable twin study with 2369 adolescents aged 16–17 years and 1067 parents, the correlation between self- and parent-rated ADHD traits was 0.37 [28]. A similar correlation between self- and parent-rated ADHD traits (*r* = 0.34) was found in the current sample, even though the response scales were different between the informants [70]. As a consequence, the phenotypic correlations between the CPRS-R and the MAAS are much lower than what is expected based on informant discrepancies only, which supports the idea that the instruments are complementary.

Further, twin designs come with standard assumptions and limitations concerning equal environments, gene–environment correlation and gene–environment interaction (G×E) [37]. The implication for the interpretation of the present findings is that the genetic effects influencing MAAS, ADHD traits and their associations could include interactions between genes and shared environment. Because G×E is thought to play an important role in ADHD [71], more complex models incorporating G×E need to be considered in future research. Further, our results on adolescent participants do not allow generalisation to different age groups, since genetic and environmental influences on individual differences can change across age [37].

The present study used an abbreviated 5-item version of MAAS, which is considered as useful as the original 15-item MAAS [12], and has the advantage that the addition to a test battery adds very little burden for the participants. However, a limitation is that MAAS represents a narrow unidimensional conceptualisation of dispositional mindfulness, although it also allows a more precise view of this particular aspect and increases the comparability of our results with most other studies on dispositional mindfulness. It has been argued that lower scores on MAAS, reflecting less perceived lapses of attention, does not necessitate high dispositional mindfulness, because mindfulness is not simply an opposite of or a lack of mindlessness or inattentiveness [72, 73]. Nevertheless, recent neuroimaging findings show that MAAS is associated with the functional connectivity of several brain regions involved in attention, emotion processing, self-processing, interoception and body awareness [13, 74] that have been associated with MBIs as well [75–79]. Because MAAS captures one component of dispositional mindfulness, it would be interesting to extend and replicate our findings using other mindfulness scales and constructs. For example, instruments based on concepts that involve not only the attention component of mindfulness, but also intention and attitude [80]. Furthermore, because all self-report mindfulness scales come with limitations [81], alternatives to self-report, like interview approaches [82], experience sampling or ecological momentary assessment [83] should be considered to increase our understanding of the many facets of dispositional mindfulness.

## Electronic supplementary material

Below is the link to the electronic supplementary material.

Supplementary material 1 (DOCX 22 kb)

## References

[1] Bishop SR, Lau M, Shapiro S, Carlson L, Anderson ND, Carmody J, Segal ZV, Abbey S, Speca M, Velting D, Devins G (2004). Mindfulness: a proposed operational definition. Clin Psychol Sci Pract.

[2] American Psychiatric Association (2013). Diagnostic and statistical manual of mental disorders.

[3] Faraone SV, Asherson P, Banaschewski T, Biederman J, Buitelaar JK, Ramos-Quiroga JA, Rohde LA, Sonuga-Barke EJS, Tannock R, Franke B (2015). Attention-deficit/hyperactivity disorder. Nat Rev Dis Primers.

[4] Buitelaar JK (2017). Optimising treatment strategies for ADHD in adolescence to minimise ‘lost in transition’ to adulthood. Epidemiol Psychiatr Sci.

[5] Crane RS, Brewer J, Feldman C, Kabat-Zinn J, Santorelli S, Williams JMG, Kuyken W (2016). What defines mindfulness-based programs? The warp and the weft. Psychol Med.

[6] Cairncross M, Miller CJ (2016). The effectiveness of mindfulness-based therapies for ADHD: a meta-analytic review. J Atten Disord.

[7] Aadil M, Cosme RM, Chernaik J (2017). Mindfulness-based cognitive behavioral therapy as an adjunct treatment of attention deficit hyperactivity disorder in young adults: a literature review. Cureus.

[8] Quaglia JT, Braun SE, Freeman SP, McDaniel MA, Brown KW (2016). Meta-analytic evidence for effects of mindfulness training on dimensions of self-reported dispositional mindfulness. Psychol Assess.

[9] Gu Y, Xu G, Zhu Y (2018). A randomized controlled trial of mindfulness-based cognitive therapy for college students with ADHD. J Atten Disord.

[10] Brown KW, Ryan RM (2003). The benefits of being present: mindfulness and its role in psychological well-being. J Personal Soc Psychol.

[11] Brown KW, West AM, Loverich TM, Biegel GM (2011). Assessing adolescent mindfulness: validation of an adapted Mindful Attention Awareness Scale in adolescent normative and psychiatric populations. Psychol Assess.

[12] Osman A, Lamis DA, Bagge CL, Freedenthal S, Barnes SM (2016). The Mindful Attention Awareness Scale: further examination of dimensionality, reliability, and concurrent validity estimates. J Personal Assess.

[13] Kong F, Wang X, Song Y, Liu J (2016). Brain regions involved in dispositional mindfulness during resting state and their relation with well-being. Soc Neurosci.

[14] Keng SL, Smoski MJ, Robins CJ (2011). Effects of mindfulness on psychological health: a review of empirical studies. Clin Psychol Rev.

[15] Keith JR, Blackwood ME, Mathew RT, Lecci LB (2017). Self-reported mindful attention and awareness, go/no-go response-time variability, and attention-deficit hyperactivity disorder. Mindfulness (NY).

[16] Black DS, Sussman S, Johnson CA, Milam J (2012). Psychometric assessment of the Mindful Attention Awareness Scale (MAAS) among Chinese adolescents. Assessment.

[17] Smalley SL, Loo SK, Hale TS, Shrestha A, McGough J, Flook L, Reise S (2009). Mindfulness and attention deficit hyperactivity disorder. J Clin Psychol.

[18] Baer RA, Smith GT, Allen KB (2004). Assessment of mindfulness by self-report: the Kentucky inventory of mindfulness skills. Assessment.

[19] Baer RA, Smith GT, Hopkins J, Krietemeyer J, Toney L (2006). Using self-report assessment methods to explore facets of mindfulness. Assessment.

[20] Flagg SA (2014) The relationships between executive functioning deficits related to ADHD and mindfulness. In: Department of Educational Psychology and Learning Systems. Florida State University, Tallahassee, Florida p 120

[21] Hoxhaj E, Sadohara C, Borel P, D’Amelio R, Sobanski E, Muller H, Feige B, Matthies S, Philipsen A (2018). Mindfulness vs psychoeducation in adult ADHD: a randomized controlled trial. Eur Arch Psychiatry Clin Neurosci.

[22] Waszczuk MA, Zavos HM, Antonova E, Haworth CM, Plomin R, Eley TC (2015). A multivariate twin study of trait mindfulness, depressive symptoms, and anxiety sensitivity. Depress Anxiety.

[23] Greven CU, Kovas Y, Willcutt EG, Petrill SA, Plomin R (2014). Evidence for shared genetic risk between ADHD symptoms and reduced mathematics ability: a twin study. J Child Psychol Psychiatry.

[24] Greven CU, Asherson P, Rijsdijk FV, Plomin R (2011). A longitudinal twin study on the association between inattentive and hyperactive-impulsive ADHD symptoms. J Abnorm Child Psychol.

[25] Burt SA (2009). Rethinking environmental contributions to child and adolescent psychopathology: a meta-analysis of shared environmental influences. Psychol Bull.

[26] Danckaerts M, Sonuga-Barke EJ, Banaschewski T, Buitelaar J, Dopfner M, Hollis C, Santosh P, Rothenberger A, Sergeant J, Steinhausen HC, Taylor E, Zuddas A, Coghill D (2010). The quality of life of children with attention deficit/hyperactivity disorder: a systematic review. Eur Child Adolesc Psychiatry.

[27] Merwood A, Greven CU, Price TS, Rijsdijk F, Kuntsi J, McLoughlin G, Larsson H, Asherson PJ (2013). Different heritabilities but shared etiological influences for parent, teacher and self-ratings of ADHD symptoms: an adolescent twin study. Psychol Med.

[28] Du Rietz E, Kuja-Halkola R, Brikell I, Jangmo A, Sariaslan A, Lichtenstein P, Kuntsi J, Larsson H (2017). Predictive validity of parent- and self-rated ADHD symptoms in adolescence on adverse socioeconomic and health outcomes. Eur Child Adolesc Psychiatry.

[29] Chen W, Zhou KX, Sham P, Franke B, Kuntsi J, Campbell D, Fleischman K, Knight J, Andreou P, Arnold R, Altink M, Boer F, Boholst MJ, Buschgens C, Butler L, Christiansen H, Fliers E, Howe-Forbes R, Gabriels I, Heise A, Korn-Lubetzki I, Marco R, Medad S, Minderaa R, Muller UC, Mulligan A, Psychogiou L, Rommelse N, Sethna V, Uebel H, McGuffin P, Plomin R, Banaschewski T, Buitelaar J, Ebstein R, Eisenberg J, Gill M, Manor I, Miranda A, Mulas F, Oades RD, Roeyers H, Rothenberger A, Sergeant J, Sonuga-Barke EJS, Steinhausen HC, Taylor E, Thompson M, Faraone SV, Asherson P (2008). DSM-IV combined type ADHD shows familial association with sibling trait scores: a sampling strategy for QTL linkage. Am J Med Genet B.

[30] Stergiakouli E, Martin J, Hamshere ML, Langley K, Evans DM, St Pourcain B, Timpson NJ, Owen MJ, O’Donovan M, Thapar A, Davey Smith G (2015). Shared genetic influences between attention-deficit/hyperactivity disorder (ADHD) traits in children and clinical ADHD. J Am Acad Child Adolesc Psychiatry.

[31] Greven CU, Rijsdijk FV, Plomin R (2011). A twin study of ADHD symptoms in early adolescence: hyperactivity-impulsivity and inattentiveness show substantial genetic overlap but also genetic specificity. J Abnorm Child Psychol.

[32] Haworth CM, Davis OS, Plomin R (2013). Twins Early Development Study (TEDS): a genetically sensitive investigation of cognitive and behavioral development from childhood to young adulthood. Twin Res Hum Genet.

[33] Van Dam NT, Earleywine M, Borders A (2010). Measuring mindfulness? An item response theory analysis of the Mindful Attention Awareness Scale. Personal Individ Differ.

[34] Conners CK, Sitarenios G, Parker JD, Epstein JN (1998). The revised Conners’ Parent Rating Scale (CPRS-R): factor structure, reliability, and criterion validity. J Abnorm Child Psychol.

[35] Diener E, Suh EM, Lucas RE, Smith HL (1999). Subjective well-being: three decades of progress. Psychol Bull.

[36] Huebner ES (1994). Preliminary development and validation of a multidimensional life satisfaction scale for children. Psychol Assess.

[37] Plomin R, DeFries JC, Knopik VS, Neiderhiser J (2013). Behavioral genetics.

[38] Boker S, Neale M, Maes H, Wilde M, Spiegel M, Brick T, Spies J, Estabrook R, Kenny S, Bates T, Mehta P, Fox J (2011). OpenMx: an open source extended structural equation modeling framework. Psychometrika.

[39] Pingault JB, Viding E, Galera C, Greven CU, Zheng Y, Plomin R, Rijsdijk F (2015). Genetic and environmental influences on the developmental course of attention-deficit/hyperactivity disorder symptoms from childhood to adolescence. Jama Psychiatry.

[40] Lehmann EL, D’Abrera HJM (1975). Nonparametrics: statistical methods based on ranks.

[41] McGue M, Bouchard TJ (1984). Adjustment of twin data for the effects of age and sex. Behav Genet.

[42] Neale MC, Miller MB (1997). The use of likelihood-based confidence intervals in genetic models. Behav Genet.

[43] Wagenmakers EJ, Farrell S (2004). AIC model selection using Akaike weights. Psychon Bull Rev.

[44] Raftery AE, Marsden PV (1995). Bayesian model selection in social research. Sociological methodology.

[45] Williams RL (2000). A note on robust variance estimation for cluster-correlated data. Biometrics.

[46] Edel MA, Holter T, Wassink K, Juckel G (2017). A comparison of mindfulness-based group training and skills group training in adults with ADHD. J Atten Disord.

[47] Biederman J, Mick E, Faraone SV (2000). Age-dependent decline of symptoms of attention deficit hyperactivity disorder: impact of remission definition and symptom type. Am J Psychiatry.

[48] Dopfner M, Hautmann C, Gortz-Dorten A, Klasen F, Ravens-Sieberer U, Group Bs (2015). Long-term course of ADHD symptoms from childhood to early adulthood in a community sample. Eur Child Adolesc Psychiatry.

[49] Eley TC (1997). General genes: a new theme in developmental psychopathology. Curr Dir Psychol Sci.

[50] Plomin R, Kovas Y, Haworth CMA (2007). Generalist genes: genetic links between brain, mind, and education. Mind Brain Educ.

[51] Middeldorp CM, Hammerschlag AR, Ouwens KG, Groen-Blokhuis MM, St Pourcain B, Greven CU, Pappa I, Tiesler CM, Ang W, Nolte IM, Vilor-Tejedor N, Bacelis J, Ebejer JL, Zhao H, Davies GE, Ehli EA, Evans DM, Fedko IO, Guxens M, Hottenga JJ, Hudziak JJ, Jugessur A, Kemp JP, Krapohl E, Martin NG, Murcia M, Myhre R, Ormel J, Ring SM, Standl M, Stergiakouli E, Stoltenberg C, Thiering E, Timpson NJ, Trzaskowski M, van der Most PJ, Wang C, Nyholt DR, Medland SE, Neale B, Jacobsson B, Sunyer J, Hartman CA, Whitehouse AJ, Pennell CE, Heinrich J, Plomin R, Davey Smith G, Tiemeier H, Posthuma D, Boomsma DI (2016). A genome-wide association meta-analysis of attention-deficit/hyperactivity disorder symptoms in population-based pediatric cohorts. J Am Acad Child Adolesc Psychiatry.

[52] Li Z, Chang SH, Zhang LY, Gao L, Wang J (2014). Molecular genetic studies of ADHD and its candidate genes: a review. Psychiatry Res.

[53] Rhee SH, Willcutt EG, Hartman CA, Pennington BF, DeFries JC (2008). Test of alternative hypotheses explaining the comorbidity between attention-deficit/hyperactivity disorder and conduct disorder. J Abnorm Child Psychol.

[54] Napoli M, Krech PR, Holley LC (2005). Mindfulness training for elementary school students. J Appl Sch Psychol.

[55] Schonert-Reichl KA, Lawlor MS (2010). The effects of a mindfulness-based education program on pre- and early adolescents’ well-being and social and emotional competence. Mindfulness (NY).

[56] Hepark S, Janssen L, de Vries A, Schoenberg PL, Donders R, Kan CC, Speckens AE (2015). The efficacy of adapted MBCT on core symptoms and executive functioning in adults with ADHD: a preliminary randomized controlled trial. J Atten Disord.

[57] Mrazek MD, Smallwood J, Schooler JW (2012). Mindfulness and mind-wandering: finding convergence through opposing constructs. Emotion.

[58] Mowlem FD, Skirrow C, Reid P, Maltezos S, Nijjar SK, Merwood A, Barker E, Cooper R, Kuntsi J, Asherson P (2016). Validation of the mind excessively wandering scale and the relationship of mind wandering to impairment in adult ADHD. J Atten Disord.

[59] Farb NA, Segal ZV, Mayberg H, Bean J, McKeon D, Fatima Z, Anderson AK (2007). Attending to the present: mindfulness meditation reveals distinct neural modes of self-reference. Soc Cogn Affect Neurosci.

[60] Garrison KA, Zeffiro TA, Scheinost D, Constable RT, Brewer JA (2015). Meditation leads to reduced default mode network activity beyond an active task. Cogn Affect Behav Neurosci.

[61] Simon R, Engstrom M (2015). The default mode network as a biomarker for monitoring the therapeutic effects of meditation. Front Psychol.

[62] Sood A, Jones DT (2013). On mind wandering, attention, brain networks, and meditation. Explore (NY).

[63] Brewer JA, Worhunsky PD, Gray JR, Tang Y-Y, Weber J, Kober H (2011). Meditation experience is associated with differences in default mode network activity and connectivity. Proc Natl Acad Sci USA.

[64] Metin B, Krebs RM, Wiersema JR, Verguts T, Gasthuys R, van der Meere JJ, Achten E, Roeyers H, Sonuga-Barke E (2015). Dysfunctional modulation of default mode network activity in attention-deficit/hyperactivity disorder. J Abnorm Psychol.

[65] Poerio GL, Sormaz M, Wang HT, Margulies D, Jefferies E, Smallwood J (2017). The role of the default mode network in component processes underlying the wandering mind. Soc Cogn Affect Neurosci.

[66] Lao SA, Kissane D, Meadows G (2016). Cognitive effects of MBSR/MBCT: a systematic review of neuropsychological outcomes. Conscious Cogn.

[67] Franklin MS, Mrazek MD, Anderson CL, Johnston C, Smallwood J, Kingstone A, Schooler JW (2017). Tracking distraction. J Atten Disord.

[68] Schoenberg PL, Hepark S, Kan CC, Barendregt HP, Buitelaar JK, Speckens AE (2014). Effects of mindfulness-based cognitive therapy on neurophysiological correlates of performance monitoring in adult attention-deficit/hyperactivity disorder. Clin Neurophysiol.

[69] Sprich SE, Burbridge J, Lerner JA, Safren SA (2015). Cognitive-behavioral therapy for ADHD in adolescents: clinical considerations and a case series. Cogn Behav Pract.

[70] Greven CU, Buitelaar JK, Salum GA (2018). From positive psychology to psychopathology: the continuum of attention-deficit hyperactivity disorder. J Child Psychol Psychiatry.

[71] Nigg J, Nikolas M, Burt SA (2010). Measured gene by environment interaction in relation to attention-deficit/hyperactivity disorder (ADHD). J Am Acad Child Adolesc Psychiatry.

[72] Chiesa A (2013). The difficulty of defining mindfulness: current thought and critical issues. Mindfulness (NY).

[73] Höfling V, Moosbrugger H, Schermelleh-Engel K, Heidenreich T (2011). Mindfulness or mindlessness?. Eur J Psychol Assess.

[74] Bilevicius E, Smith SD, Kornelsen J (2018). Resting-state network functional connectivity patterns associated with the Mindful Attention Awareness Scale. Brain Connect.

[75] Mitchell JT, Zylowska L, Kollins SH (2015). Mindfulness meditation training for attention-deficit/hyperactivity disorder in adulthood: current empirical support, treatment overview, and future directions. Cogn Behav Pract.

[76] Bachmann K, Lam AP, Philipsen A (2016). Mindfulness-based cognitive therapy and the adult ADHD brain: a neuropsychotherapeutic perspective. Front Psychiatry.

[77] Tang YY, Holzel BK, Posner MI (2015). The neuroscience of mindfulness meditation. Nat Rev Neurosci.

[78] Tang YY, Ma Y, Wang J, Fan Y, Feng S, Lu Q, Yu Q, Sui D, Rothbart MK, Fan M, Posner MI (2007). Short-term meditation training improves attention and self-regulation. Proc Natl Acad Sci USA.

[79] Tang YY, Lu Q, Fan M, Yang Y, Posner MI (2012). Mechanisms of white matter changes induced by meditation. Proc Natl Acad Sci USA.

[80] Shapiro SL, Carlson LE (2009). The art and science of mindfulness: integrating mindfulness into psychology and the helping professions.

[81] Park T, Reilly-Spong M, Gross CR (2013). Mindfulness: a systematic review of instruments to measure an emergent patient-reported outcome (PRO). Qual Life Res.

[82] Grossman P (2011). Defining mindfulness by how poorly I think I pay attention during everyday awareness and other intractable problems for psychology’s (re)invention of mindfulness: comment on Brown et al. (2011). Psychol Assess.

[83] Davidson RJ, Kaszniak AW (2015). Conceptual and methodological issues in research on mindfulness and meditation. Am Psychol.

