# Supplementary material for: Genetic and environmental aetiologies of associations between dispositional mindfulness and ADHD traits: a population-based twin study
Source: Eur Child Adolesc Psychiatry. 2019 Feb 13;28(9):1241–51. doi: 10.1007/s00787-019-01279-8 (PMC6751144; doi:10.1007/s00787-019-01279-8)
Supplement: Supplementary file 1 — Supplementary material 1 (DOCX 22 kb) [file 787_2019_1279_MOESM1_ESM.docx]

Genetic and Environmental Aetiologies of Associations Between Dispositional Mindfulness and ADHD Traits: a Population-Based Twin Study

European Child & Adolescent Psychiatry

Nienke M. Siebelink*, Philip Asherson, Elena Antonova , Susan M. Bögels, Anne E. Speckens, Jan K. Buitelaar & Corina U. Greven

*Address for correspondence: Karakter, Child and Adolescent Psychiatry, University Center, Reinier Postlaan 12, 6525 GC Nijmegen, the Netherlands. Telephone: 0031 24 351 2222.

E-mail address: [n.siebelink@karakter.com](mailto:n.siebelink@karakter.com)

Table 1. Sample representativeness

|  | N twins (individuals) | % White | % Mothers with A-levels or higher | % Mothers employed | % Fathers employed | % Female | % MZ |
| --- | --- | --- | --- | --- | --- | --- | --- |
| UK census^1^ | - | 93% | 32% | 49% | 89% | - | - |
| TEDS first contact^2^ | 27870 | 92% | 36% | 43% | 92% | 50% | 33% |
| TEDS 16-year wave (present sample)^3^ | 2830 | 93% | 38% | 45% | 93% | 58% | 37% |

*Note.* ^1^UK data from the 2000 General Household Survey which provides appropriate comparisons for the TEDS twins who were born between 1994-96. ^2^TEDS sample at first contact with twins at around 18 months. ^3^Demographics only for individuals in the 16-year wave who had data for the present manuscript. The sample remains representative of the UK population and first contact sample in terms of key demographic indexes. A-levels = national educational exam taken around age 18 years in the UK. MZ = monozygotic twins.

Table 2. Descriptives

| Twin 1 |  |  |  |  |  |  |  |  |  |  |
| --- | --- | --- | --- | --- | --- | --- | --- | --- | --- | --- |
|  |  | Female |  |  |  | Male |  |  |  |  |
|  | N (individuals) | Mean | SD (range) |  | N (individuals) | Mean | SD (range) |  | Skew | Cronbach’s alpha |
| MAAS | 656 | 9.14 | 4.28 (0-20) |  | 417 | 8.57 | 4.36 (0-20) |  | -0.16 | 0.76 |
| INATT (p) | 654 | 3.25 | 4.08 (0-21) |  | 427 | 4.90 | 5.44 (0-26) |  | 1.75 | 0.90 |
| HYP-IMP (p) | 654 | 2.33 | 3.10 (0-21) |  | 427 | 2.38 | 3.38 (0-27) |  | 2.63 | 0.78 |
| Life satisfaction | 642 | 4.63 | 0.62 (2-6) |  | 425 | 4.67 | 0.59 (2-6) |  | -0.72 | 0.86 |
| INATT (s) | 656 | 29.59 | 7.90 (9-58) |  | 416 | 30.04 | 8.27 (9-61) |  | 0.06 | 0.89 |
| HYP/IMP (s) | 656 | 28.74 | 9.36 (9-60) |  | 416 | 29.04 | 8.84 (9-58) |  | -0.05 | 0.90 |
| Twin 2 |  |  |  |  |  |  |  |  |  |  |
|  |  | Female |  |  |  | Male |  |  |  |  |
|  | N (individuals) | Mean | SD (range) |  | N (individuals) | Mean | SD (range) |  | Skew | Cronbach’s alpha |
| MAAS | 649 | 9.28 | 4.45 (0-23) |  | 418 | 8.53 | 4.35 (0-21) |  | 0.064 | 0.76 |
| INATT (p) | 655 | 3.93 | 4.51 (0-27) |  | 427 | 3.87 | 4.66 (0-26) |  | 1.77 | 0.91 |
| HYP-IMP (p) | 654 | 2.42 | 3.27 (0-25) |  | 426 | 2.21 | 3.04 (0-22) |  | 2.44 | 0.78 |
| Life satisfaction | 611 | 4.62 | 0.60 (2-6) |  | 452 | 4.69 | 0.61(2-6) |  | -0.61 | 0.86 |
| INATT (s) | 653 | 30.06 | 7.56 (9-57) |  | 417 | 29.51 | 7.60 (9-50) |  | -0.07 | 0.87 |
| HYP/IMP (s) | 653 | 29.00 | 9.06 (9-58) |  | 417 | 28.12 | 8.47 (9-60) |  | -0.04 | 0.89 |

*Note.* HYP/IMP = hyperactivity-impulsivity; INATT = inattentiveness; MAAS = Mindful Attention Awareness Scale. Descriptives based on raw data (untransformed and unregressed). Results shown separately for one randomly selected twin per pair (twin 1) and the co-twins (twin 2). Descriptives were similar for twin 1 and twin 2. (p) = Parent reported ADHD traits on the Revised Conners’ Parent Rating Scale. (s) = Self-reported ADHD traits on the Strength and Weaknesses of ADHD Symptoms and Normal behaviour questionnaire.
